# *Phellinus noxius*: molecular diversity among isolates from Taiwan and its phylogenetic relationship with other species of *Phellinus* based on sequences of the ITS region

**DOI:** 10.1186/s40529-017-0162-1

**Published:** 2017-01-16

**Authors:** Jyh-Nong Tsai, Pao-Jen Ann, Ruey-Fen Liou, Wen-Hsui Hsieh, Wen-Hsiung Ko

**Affiliations:** 1 Division of Plant Pathology, Taiwan Agricultural Research Institute, Wufeng, Taichung, Taiwan; 2 Department of Plant Pathology and Microbiology, National Taiwan University, Taipei, Taiwan; 3 Department of Plant Pathology, National Chung Hsing University, Taichung, Taiwan

**Keywords:** Deletion, Insertion, ITS sequence, Nucleotide variation, Sequence length, Single nucleotide polymorphism

## Abstract

**Background:**

Analysis of phylogenetic relationship of 91 isolates of *Phellinus noxius* obtained from 46 plant species in Taiwan did not show distinct grouping based on ITS sequences.

**Results:**

However, the ITS nucleotides showed 20 different kinds of variations including single nucleotide polymorphisms, deletion and insertion in ITS1 and ITS2, but none in 5.8 S. The Taiwanese isolates of *P. noxius* were dividable into long (type L), median (type M) and short (type S) groups based on ITS sequence length. Two isolates with identical ITS sequence belonged to types L. Type M with 72 isolates was further divided into 33 subtypes, while types S with 17 isolates was further divided into two subtypes.

**Conclusion:**

Phylogenetic analysis of ITS sequences among *Phellinus* species showed that isolates of *P. noxius* were in the same clade distinctly separated from other *Phellinus* species.

## Background

Brown root rot caused by *Phellinus noxius* (Corner) G. H. Cunn. is widespread among tropical countries in Southeast Asia, Africa, Oceania, Central America and the Caribbean (Pegler and Waterston 1968). In China, it has been reported from the tropical Hainan Island (Tai 1979). In Japan, it was found on the subtropical island of Okinawa (Abe et al. 1995). The pathogen attacks more than 120 species of fruit and ornamental trees in both topical and subtropical districts in Taiwan (Ann et al. 1999; Chang and Yang 1998). Among the approximately 200 plant species listed as hosts of *P. noxius* in the world, about half of them were reported for the first time from Taiwan (Ann et al. 2002). Even though the fungus lacks air-borne spores for efficient dissemination, it is very widespread and occurs on so many kinds of hosts at very different geographic locations on the island of Taiwan (Ann et al. 2002). It is, therefore, conceivable that *P. noxius* may be an ancient residence of the island where diverse isolates of this fungus may have existed. There are very few morphological characters in *P. noxius* available for testing this hypothesis because the fungus rarely produces basidiocarps on diseased trees in the fields (Ann et al. 1999; Chang 1995, 1996).

In this study, molecular variation in the ITS (ITS1, 5.8S and ITS2) region among isolates of *P. noxius* from Taiwan was investigated and compared with the ITS sequences reported from other countries available in the GenBank. We also investigated the ITS phylogenetic relationship of *P. noxius* with other species of *Phellinus*. Details of the study are reported herein.

## Methods

### Isolation and storage of the pathogen

Main roots of trees showing quick or slow decline symptoms (Ann et al. 2002) were exposed and examined. Those showing typical brown discoloration were cut and brought back to the laboratory. Small pieces (5 × 2 × 1 mm) of tissue were obtained from the advancing margins of the diseased roots, surface-sterilized with 0.5% NaClO for 1 min, plated on potato dextrose agar (PDA) supplemented with 100 ppm streptomycin sulfate and 10 ppm benomyl for inhibition of growth of bacteria and other fungi, and incubated at room temperature (24–30 °C). Fungal mycelia growing from diseased tissue were transferred to 2% water agar. Single-hyphal tips obtained from the fungus growing on water agar were cultured on PDA and stored in sterile distilled water in test tubes at room temperature (Boesewinkle 1976; Ko 2003). From each diseased tree only one isolate was saved for the study. The cultures were identified as *P. noxius* based on the production of brown colonies with irregular dark brown zone lines on PDA and formation of arthrospores and trichocysts (Ann and Ko 1992).

### DNA extraction, amplification and sequencing

Each isolate of *P. noxius* was grown on cellophane placed on PDA (Ko et al. 2011). After incubation at 25 °C for 10 days, mycelia were harvested, lyophilized and stored at −20 °C until use. About 20 mg lyophilized mycelia were ground in liquid nitrogen and used for extraction of DNA using the genomic DNA extraction kit (GenMark Technology Co., Taichung, Taiwan). The ITS (ITS1-5.8S-ITS2) region was amplified with primer pair of ITS4 and ITS5 (White et al. 1990). The 25 μl reaction mixture consisting of 0.2 μg template DNA, 0.2 μM each primer, 200 μM each dNTP, 2 μl 2X polymerase chain reaction (PCR) buffer and 1.0 U ZyM Taq DNA polymerase (Zymeset, Taiwan) was subjected to thermal cycling in a Perkin-Elmer Thermal Cycler 9700 (Perkin-Elmer Applied Biosystem, USA). Cycling conditions for amplification were an initial denaturation at 94 °C for 3 min, followed by 35 cycles at 94 °C for 45 s, 50 °C for 45 s, 72 °C for 45 s, and a final elongation at 72 °C for 7 min. The PCR products were electrophoresed on a 1.5% agarose gel. Direct sequencing of the PCR products was performed by the Seeing Bioscience Company (Taipei, Taiwan), using ITS4, ITS5 (White et al. 1990), PN-5.8S-1 (5′-GCA GCG AAA TGC GAT AAG TA-3′), or PN-5.8S-2 (5′-CAT GAC ACT CAA ACA GGC AT-3′) as the primer. The sequences of ITS region obtained from the sequencing process were assembled, trimmed and edited using the Vector NT1 software v. 10.0 (InforMax Inc., USA). The sequence of ITS tail was determined using the ITS 2 annotation tool (Keller et al. 2009). The polymorphic portions were marked by IUPAC ambiguity codes. The ITS sequences of 36 isolates of *P. noxius*, representing all ITS types found in Taiwan, were submitted to NCBI (National Center for Biotechnology Information; http://www.ncbi.nlm.mih.gov).

### Phylogenetic analysis

The ITS sequences of 91 isolates of *P. noxius* from Taiwan were analyzed in order to understand the phylogenetic relationship among these isolates. Multiple alignments and minor adjustments of the sequences of these isolates were performed using clustal X 1.81 (Thompson et al. 1977) followed by BioEditor software. Sequence alignment was deposited at TreeBase (http://purl.org/phylo/treebase/phylows/study/TB2:S16384). Phylogenetic relationships were analyzed using the Philip 3.67 software (Phylogeny Inference Package, Version 3.67) and the neighbor joining program with 1000 bootstrap replicates. The program of Treeview was used to view phylogenetic trees.

In order to study the phylogenetic relationship between isolates of *P. noxius* from Taiwan and other countries and other *Phellinus* species, the ITS sequences of all *Phellinus* species in the GenBank were retrieved. A total of 58 isolates belonging to 39 species of *Phellinus* was obtained and used for phylogenetic analysis (Table 1). The ITS types L, M and S divided based on ITS length were used as local strains for analysis. The method described above was used for phylogenetic relationship analysis.

**Table 1:** List of taxa of *Phellinus noxius* and related species from GenBank used for phylogenetic analysis

Species	Geographic origin^a^	Strain no.^b^	ITS sequence length (bp)^c^	Accession no.
1. * Phellinus alni*		TW322	610	AY340041
2. * P. badius*		CBS 449.76	663	AY558609
3. * P. baumii*		MPNU 7006	711	AF200231
4. * P. bicuspidatus*		KCTC 6651	621	AY558610
5. * P. calcitratus*			584	JF894115
6. * P. chrysoloma*			644	AF055370
7. * P. cinereus*		05-37	620	AM931248
8. * P. conchatus*		CBS 167.29	708	AY558614
9.* P. ferrugineovelutinus*		CBS 218.48	542	AY558618
10. * P. gilvus*		ATCC26729	613	AF250932
11. * P. hartigii*		CBS 162.30	692	AY558621
12. *P. h ippophaeicola*		CBS 252.50	705	AY558622
13. * P. igniarius*		CFMR 5698	609	AY558623
* P. igniarius*		KCTC6228	598	AF056192
14. * P. igniarius var. trivialis*		CBS 512.63	596	AY558624
15. * P. johnsonianus*		ATCC60051	702	AF250931
16. * P. laevigatus*		CFMR 5640	588	AY558626
17.* P. linteus*		MPNU 7002	670	AF200228
18. * P. lundellii*		CBS 540.72	605	AY558630
19. *P. merrillii*		PM950703-1	707	EU035310
20. *P. nigricans*		CBS 213.48	611	AY558631
* P. nigricans*		H6002112	621	GQ383726
21. * P. noxius*	Taiwan	PN72.1	613-L	JQ003239
* P. noxius*	Taiwan	PNP1.2	609-M	JN836341
* P. noxius*	Taiwan	PN29.1	609-M	JN836344
* P. noxius*	Taiwan	PNA4.1	609-M	JN836346
* P. noxius*	Taiwan	PN5.2	608-M	JQ003233
* P. noxius*	Taiwan	PNP4.2	607-M	JQ029276
* P. noxius*	Taiwan	PN22.1	601-S	EF065630
* P. noxius*		CBS170.32	601-S	EF065631
* P. noxius,*	Japan	Tf566	601-S	JQ003238
* P. noxius*	Malaysia	FRIM638	610-M	HQ400698
* P. noxius*	Malaysia	FRIM618	602-S	HQ400699
* P. noxius*	Malaysia	FRIM613	602-S	HQ400700
* P. noxius*	Malaysia	FRIM551	603-S	HQ400702
* P. noxius*	Malaysia	FRIM154	601-S	HQ400703
* P. noxius*	Malaysia	FRIM147	599-S	HQ400704
* P. noxius*	India	–	608-M	AB639022
22. *P. occidentalis*		CBS 196.55	706	AY558634
23. *P. pachyphloeus* = *Inonotus pachphloeus*		CBS 193.37	571	AY558635
24. *P. pini*		ATCC12240	635	AF250930
25. *P. pini* var. *cancriformans*		IMSNU 32031	636	AF200242
26. *P. pomaceus*		25	599	FR686572
27. * P. populicola*		CBS 638.75	599	AY558638
28. *P. punctatus*		CBS 386.66	649	AY558640
29. *P. repandus*		CBS 616.89	658	AF534076
30. *P. rhabarbarinus*		CBS 282.77	714	AY558642
31. *P. ribis f. ulicis*		CBS 579.50	653	AY558644
32. *P. rimosus*		MDJCBS86	608	DQ103885
33. *P. robustus*		KCTC 6657	679	AY558645
34. *P. senex*		CBS 442.76	578	AY558647
35. *P. spiculosus*		KTCC 6658	641	AY558648
36. *P. tremulae*		CBS 123.40	595	AY558650
37. *P. tropicalis*		CBS 617.89	636	AF534077
38. *P. tuberculosus*		CBS 171.32	600	AY558652
39. *P. weirii*		CNU 6017	620	AF251438

^a^The country where *P. noxius* was isolated

^b^ *CBS* Centraalbureau voor Schimmelcultures, *NPMU* National Programme Management Unit, *KCTC* Korean Collection for Type Cultures, *ATCC* American Type Culture Collection, *CFMR* Colegiul Fizicienilor Medicali din România, *FRIM* Forest Research Institute Malaysia, *IMSNU* Institute of Microbiology, Seoul National University, *CNU* Collection of Newcastle University

^c^L: ITS type L; M: ITS type M; S: ITS type S

## Results

### Phylogenetic relationship among Taiwanese isolates of P. noxius

A total of 91 isolates of *P. noxius* was obtained from 46 species of plants distributed in different geographic locations in Taiwan from 1991 to 2009 (Table 2). Analysis of the phylogenetic relationship of these Taiwanese isolates did not show distinct grouping based on ITS sequences. The bootstrap values on the branches were very low and were all below 50% (data not shown) with accession number JN836346-JQ003229 (Tables 1, 2).

**Table 2:** List of hosts, locations, ITS information, GenBank accession no. of strains of *Phellinus noxius* from Taiwan used in the study

Scientific name (common name)	Isolate	Location	Year of isolation	GenBank accession no.	Sequence ITS1/5.8S/ITS2 (bp)	ITS type
1. * Annona squamosa* (custard apple)	PNA4.1	Taitung County	1996	JN836346	609	M6
2.* Araucaria cunninghamii* (hook pine)	PN29.1	Taichung City	2004	JN836344	609	M4
* A. cunninghamii*	PN30.1	Taichung City	2004		609	M4
3. *Averrhoa carambola* (carambola)	PNS1.1	Tainan City	1992		607	M32
4. *Bauhinia* × hybrid (butterfly tree)	PN40.2	Changhua County	2005	JQ003235	606	M33
5. *Bauhinia variegata* (orchid tree)	PN7.1	Nantou County	1996		609	M6
* B. variegata*	PN35.1	Hualian County	2005	JN836349	609	M9
* B. variegata*	PN35.2	Hualian County	2005		609	M9
6. *Calocedrus formosana* (Taiwan incense cedar)	PN70.2	Taichung City	2009	JQ003232	608	M23
7. *Casuarina equisetifolia* (ironwood tree)	PN22.1	Nantou County	1998	EF065630.1	601	S1
8. *Cinnamomum kotoensis* (botel tobago cinnamon tree)	PN74.2	Taitung County	2009		608	M24
* C. kotoensis*	PN74.1	Taitung County	2009	JQ029271	608	M27
9. *C. osmophloeum* (Taiwan cinnamon)	PN50.1	Nantou County	2006		601	S1
* C. osmophloeum*	PN51.1	Nantou County	2006		608	M24
10. *Cinnamomun camphora* (camphor)	PN32.1	Taichung City	2005		608	M24
* C. camphora*	PN32.2	Taichung City	2005		608	M24
* C. camphora*	PN94001.1	Nantou County	2005		607	M32
* C. camphora*	94001.2	Nantou County	2005		607	M32
11. *Citrus limon* (lemon)	PNC1.1	Tainan City	2003		609	M1
* C. limon*	PNC1.2	Tainan City	2003		609	M1
* C. limon*	PNC4.1	Chiayi County	2006		607	M32
12. *Delonix regia* (flame tree)	PN37.1	Hualian County	2005		601	S1
* D. regia*	PN37.2	Hualian County	2005	JQ029275	607	M31
* D. regia*	PN42.1	Hualian County	2005		601	S1
13. *Dimocarpus. longan* (longan)	PNLn5.1	Tainan City	1992	JQ003236	601	S1
* D. longan*	PNLn9.2	Changhua County	1998	JQ003226	608	M17
* D. longan*	PNLn10.1	Tainan City	2003		609	M6
* D. longan*	PNLn10.2	Tainan City	2003	JQ003222	609	M13
* D. longan*	PNLn14.2	Changhua County	2006		601	S1
14. *Diospyros kaki* (persimmon)	PNPe1.1	Chiayi County	1991		609	M1
15. *Duranta repens* (creeping sky flower)	PN3.1	Nantou County	1996	JQ003231	608	M22
16. *Eriobotrya japonica* (loquat)	PNLo3.1	Taitung County	1997		601	S1
* E. japonica*	PNLo5.1	Taitung County	2009		609	M1
17. *Eucalyptus citriodora* (lemon gum eucalyptus)	PN6.1	Nantou County	1996		608	M22
18. *Ficus microcarpa* (small-leafed banyan)	PN21.1	Miaoli County	2003		608	M24
* F. microcarpa*	PN21.2	Miaoli County	2003		608	M24
* F. microcarpa*	PN12.1	Taichung City	1996	JQ029274	607	M30
* F. microcarpa*	PN26	Nantou County	2003		608	M24
* F. microcarpa*	PN28.2	Taichung City	2004		608	M19
* F. microcarpa*	PN49.2	Taichung City	2005	JQ003227	608	M18
* F. microcarpa*	PN57.1	Taichung City	2005		609	M7
* F. microcarpa*	PN75.1	Taichung City	2009		607	M30
* F. microcarpa*	PN76.1	Taichung City	2009		609	M4
19. * Ficus pumila* var. *awkeotsang* (jellyfig)	PN10.1	Chiayi County	1991	JQ029272	608	M28
20. * F. religiosa* (botree fig)	PN90.1	Taichung	2009	JN836342	609	M2
21. *Juniperus chinensis* var. kaizuka (dragon juniper).	PN65.1	Nantou County	2007		609	M4
22. *Kigelia pinnata* (sausage tree)	PN14.1	Nantou County	1998	JN836348	609	M8
23. *Koelreuteria henryi* (flame gold-rain tree)	PN94.1	Taichung City	2009	JQ003237	601	S2
*K. henryi*	PN33.2	Hualian County	2005	JQ003223	609	M14
*K. henryi*	PN41.1	Hualian County	2005		609	M14
*K. henryi*	PN41.2	Hualian County	2005		609	M14
24. *Liquidambar formosana* (maple)	R9218	New Taipei city	1992		608	M15
25. *Litchi chinensis* (litchi)	PNL2.1	Chiayi County	1992	JN836347	609	M7
*L. chinensis*	PNL2.2	Chiayi County	1992		601	S1
*L. chinensis*	PNL5.1	Kaohsiung City	2003	JQ029273	607	M29
*L. chinensis*	PNL5.2	Kaohsiung City	2003		607	M29
26. *Mangifera indica* (mango)	PNM4.1	Changhua County	2009		609	M6
27. *Melaleuca bracteata* ‘Revolution Gold’ (white cloud tree)	PN73.2	Taichung City	2009		609	M6
*M. bracteata* ‘Revolution Gold’	PN73.1	Taichung City	2009		609	M6
28. *Murraya paniculata* (orange jasmine)	PN5.1	Nantou County	1996		608	M24
*M. paniculata*	PN5.2	Nantou County	1996	JQ003233	608	M24
*M. paniculata*	PN25.1	New Taipei city	2004		601	S1
*M. paniculata*	PN25.2	New Taipei city	2004		601	S1
29. *Oncidium* Gower Ramsey	PN44	Yungling County	2005		601	S1
30. *Osmanthus fragrans* (osmanthus)	PN140.1	Changhua County	2009	JQ003221	609	M12
31. *Psidium guajava* (guava)	PN98007	Kaohsiung City	2009	JQ029270	608	M26
32. *Podocarpus macrophyllus* (long-leaved podocarpus)	PN98.3	Taichung City	2009		601	S1
33. *Prunus armeniaca* (apricot)	PN72.1	Taichung City	2009	JQ003239	613	L1
*P. armeniaca*	PN72.2	Taichung City	2009		613	L1
34. *Prunus campanulata* (Taiwan cherry)	PN71.1	Taichung City	2009	JN386350	609	M10
*P. campanulata*	PN71.2	Taichung City	2009		609	M10
35. *Prunus mume* (plum)	PNP1.2	Kaohsiung City	1991	JN836341	609	M1
*P. mume*	PNP2.1	Nantou County	1996	JN836345	609	M5
36. *Prunus persica* (peach)	PNP5.1	Nantou County	1999	JQ003228	608	M19
*P. persica*	PNP10.1	Changhua County	2005	JQ003225	608	M16
37. *Pterocarpus indicus* (rose wood)	PN104.1	Taichung City	2009	JQ003220	609	M11
38. *Pyrus pyrifolia* (pear)	PNP4.1	Miaoli County	2003		601	S1
*P. pyrifolia*	PNP4.2	Taichung City	1998	JQ029276	607	M32
*P. pyrifolia*	PNP9.1	Nantou County	2004	JN836343	609	M3
39. *Schinus terebinthifolius* (Brazilian peppertree)	PN48.1	Taichung City	2005		601	S1
*S. terebinthifolinus*	PN48.2	Taichung City	2005		601	S1
40. *Spathodea campanulata* (African tulip tree)	PN147	Changhua County	2009		601	S1
41. *Sterculia nobilis* (ping-pong)	PN17.1	Nantou County	1999	JQ003224	608	M15
*S. nobilis*	PN84.1	Taichung City	2009		607	M32
*S. nobilis*	PN124.1	Taichung City	2009		609	M6
42. *Syzygium samarangense* (wax apple)	PNW1.1	Chiayi County	1991	JQ003234	608	M25
43. *Terminalia catappa* (Indian almond)	PN2.1	Chiayi County	1996		609	M4
44. *T. catappa*	PN63.1	Changhua County	2007		607	M32
45. *Toona sinensis*, *Cedrela sinensis* (Chinese cedar)	PN64.1	Taichung City	2007	JQ003230	608	M21
46. *Vitis vinifera* (grape)	PNG1.1	Nantou County	1999		609	M6
*Zizyphus mauritiana* (Indian jujube)	PNZ2.1	Kaohsiung City	2001	JQ003229	608	M20

### Nucleotide variation in ITS region among Taiwanese isolates of *P. noxius*

The examination of ITS nucleotide variation revealed the existence of 20 different kinds of variants, designated as V1 to V20 in ITS1 and ITS2 but not 5.8S in the 91 Taiwanese isolates of *P. noxius* obtained in this study (Table 3) . The variation included insertion, deletion and single nucleotide polymorphism. Some isolates showed single nucleotide polymorphism among chromosomes in the same isolate.

**Table 3:** Nucleotide variation in ITS detected among isolates of *Phellinus noxius* in Taiwan

Kind of variant	Sequence position	Nucleotide variation		
		Single nucleotide polymorphism	Deletion	Insertion
ITS1				
V1/V1*	19	G, A/R		
V2	31	T, C		
V3/V3*	32	G, C/S		
V4	114	G, C		
V5	116–117			GGAGAG
V6	117–118	TG, AT		
V7	125–126	TC, AT		
V8/V8*	129	T, A/C		
V9	135–142		ATTTATTC	
V10	152	A, G		
V11	168	C, T		
V12	193		A	
V13	197	T, C		
ITS2				
V14/V14*	420	T, C/Y		
V15/V15*	442	G, A/R		
V16/V16*	469	A, G/R		
V17/V17*	546	A, G/R		
V18/V18*	593	C, G/S		
V19	594–595		AC	
V20	600–601			C

*The variants with an asterisk symbol represent isolates with single nucleotide polymorphisms among chromosomes in the same isolate

### Grouping based on ITS sequence length

The examination of ITS nucleotide variation also revealed the possible division into three distinct groups based on sequence length among the 91 isolates of *P. noxius* from Taiwan (Table 4). Isolates with long sequence of 613 bp were termed type L. Only two isolates belonged to this type. Isolates with median sequence length of 606–609 bp were termed type M. The majority of the Taiwanese isolates with a total of 72 isolates belonged to this type. Type M was further divided into 33 subtypes based on single nucleotide polymorphisms, single nucleotide deletion (V12), double nucleotide deletion (V19) and single nucleotide insertion (V20) (Tables 3, 4) . Isolates with short sequence of 601 bp were termed type S. Type S was further divided into two subtypes as a result of a single nucleotide polymorphism at position 114. Seventeen isolates belonged to this type.

**Table 4:** ITS types and subtypes among *Phellinus noxius* isolates from Taiwan

ITS type and subtype	ITS sequence length (bp)	Nucleotide variation	Representative isolate (total no.)
Type L			
L1	613	V1, V3, V5, V15, V16, V19	PN72.1 (2)
Type M			
M1	609		PNP1.2 (5)
M2	609	V16	PN90.1 (1)
M3	609	V17	PNP9.1 (1)
M4	609	V18	PN29.1 (5)
M5	609	V1, V3	PNP2.1 (1)
M6	609	V14, V15	PNA4.1 (8)
M7	609	V15, V16	PNL2.1 (2)
M8	609	V3, V14, V15	PN14.1 (1)
M9	609	V1, V3, V16, V17	PN35.1 (2)
M10	609	V1, V3, V11, V15, V17	PN71.1 (2)
M11	609	V15*, V18*	PN104.1 (1)
M12	609	V1, V15*, V16	PN140.1 (1)
M13	609	V14*, V15, V17*	PNLn10.2 (1)
M14	609	V1*, V3*, V15*, V16*	PN33.2 (3)
M15	608	V3, V12	PN17.1 (2)
M16	608	V3, V12, V17	PNP10.1 (1)
M17	608	V10, V12, V15, V16	PNLn9.2 (1)
M18	608	V1, V3, V11, V12	PN49.2 (1)
M19	608	V1, V3, V8, V11, V12, V16	PNP5.1 (2)
M20	608	V1, V3, V6, V11, V12, V15	PNZ2.1 (1)
M21	608	V1, V2, V3, V11, V12, V15, V16	PN64.1 (1)
M22	608	V1, V2, V3, V8, V11, V12, V13, V16	PN3.1 (2)
M23	608	V1, V2, V3, V6, V7, V11, V12, V15, V16	PN70.2 (1)
M24	608	V15, V16, V19, V20	PN5.2 (9)
M25	608	V1, V3, V15, V16, V19, V20	PNW1.1 (1)
M26	608	V3, V12, V15*, V16*	PN98007 (1)
M27	608	V15, V16*, V19, V20	PN74.1 (1)
M28	608	V1, V3*, V15, V19, V20	PN10.1 (1)
M29	607	V19	PNL5.1 (2)
M30	607	V16, V19	PN12.1 (2)
M31	607	V15, V16, V19	PN37.2 (1)
M32	607	V1, V3, V15, V16, V19	PNP4.2 (7)
M33	606	V1, V2, V3, V6, V7, V11, V12, V15, V16, V19	PN40.2 (1)
Type S			
S1	601	V9	PNLn5.1 (16)
S2	601	V4, V9	PN94.1 (1)

*The variants with an asterisk symbol represent isolates with single nucleotide polymorphisms among chromosomes in the same isolate

Isolates of *P. noxius* from GenBank fitted or nearly fitted the M or S ITS types (in Taiwan; Table 1). Isolate CBS170.32 of unknown origin belonged to type S, so was the Japanese isolate. The isolate from India belonged to type M. Among the six isolates from Malaysia, isolate FRIM154 fitted the type S and isolate FRIM 638 nearly fitted the type M with 1 bp more than the Taiwanese type M. Isolates FRIM 618, FRIM 613 and FRIM 551 nearly fitted type S with 1–2 bp more than the Taiwanese type S, while isolates FRIM 147 was also close to type S with 2 bp less than the Taiwanese type S. However no isolates from other countries were founded to fit the Taiwanese type L in this study.

### Relation between ITS types and hosts and locations from where *P. noxius* was found in Taiwan

**Fig. 1:**
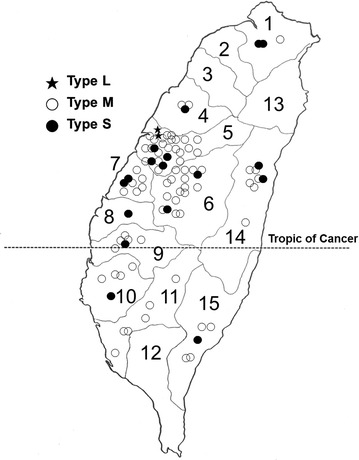
Distribution of different ITS types of *Phellinus noxius* from Taiwan. Number referred to *1* New Taipei City, *2* Taoyuan County, *3* Hsinchu County, *4* Miaoli County, *5* Taichung City, *6* Nantou County, *7* Changhua County, *8* Yunlin County, *9* Chiayi County, *10* Tainan City, *11* Kaohsiung City, *12* Pingtung County, *13* Yilan County, *14* Hualian County, *15* Taitung County

Type L was detected only in Taichung City (Fig. 1). Type M was found in three cities and seven counties, while type S was found in two cities and eight counties. *P. noxius* was not found in Yilan County, Taoyuan County, Hsinchu Tounty and Pingtung County during this study.

Subtype S1 was found on 12 plant species located in three cities and seven counties, while subtype S2 was found only on flame gold-rain tree in Taichung City (Table 2). Other isolates found on flame gold-rain tree in Hualian County belonged to subtype M14. This study also revealed that isolates of *P. noxius* obtained from the same plant species in the same location may belong to different subtypes. In Taichung City, isolates of *P. noxius* found on small-leafed banyan consisted of subtypes M4, M7, M18, M19 and M30. Similarly, isolates obtained from longan in Tainan City contained subtypes S1, M6 and M13. It was also found that isolates obtained from the same host in different locations may belong to the same subtype. For examples, subtype S1 on longan was found in Tainan City and Changhua County, while subtype M24 on small-leafed banyan was found in Miaoli County and Nantou County. Isolates obtained from different hosts in different locations may also belong to the same subtype. For examples, subtype M1 was found on lemon in Tainan City and on persimmon in Chiayi County, while subtype M6 was found on custard apple in Taitung County and on orchid tree in Nantou County.

### Phylogenetic analysis based on ITS sequences among *Phellinus* species

**Fig. 2:**
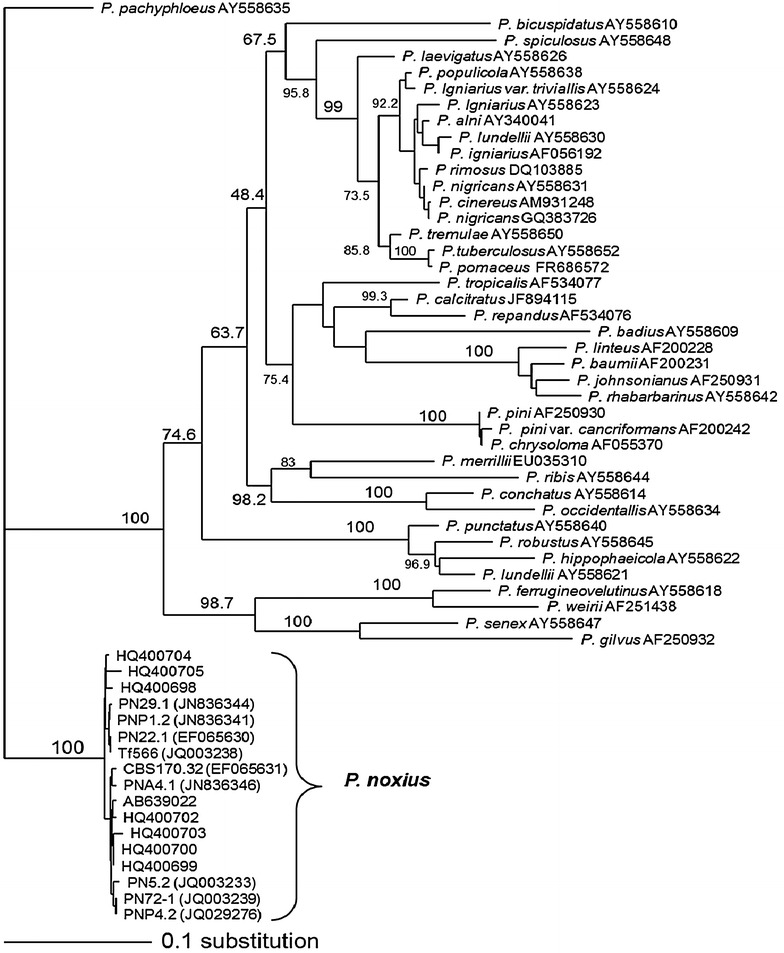
The (unrooted) distance tree of phylogenic relationship of 17 *Phellinus noxius* isolates and other 38 species in *Phellinus* spp. based on the internal transcribed spacer (ITS1-5.8Sr DNA-ITS2) region of nuclear ribosomal DNA. Branch lengths and boot strap (1000 replicates) were displayed in the distance tree by using neighbor-joining and bootstrap methods of Philip 3.67 software. The distance bar is corresponded to 10 substitutions per 100 nucleotide sites

The ITS sequences of 58 isolates belonging to 39 species of *Phellinus* retrieved from GenBank and seven *P. noxius* isolates representing type L, type M and type S of ITS sequences from Taiwan were used in the analysis of the phylogenetic relationship among *Phellinus* species. The result showed that all the isolates of *P. noxius* including isolates from Taiwan and other countries were in the same clade with 100% bootstrap support (Fig. 2). The sequence similarity between *P. noxius* and other *Phellinus* species was less than 85%. The species most closely related to *P. noxius* was *P. pachphloeus* with 83% similarity, whereas the most distant species was *P. badius* with only 67% similarity.

## Discussion

Results from this study showed that the isolates of *P. noxius* from Taiwan can be divided into type L, type M and type S based on ITS sequence length. From 1991 to 2009, 2 type L isolates, 82 type M isolates and 17 type S isolates were found on 46 plant species in Taiwan (Table 2). To our best knowledge, this is the first report of division of isolate from the same fungal species into different groups based on ITS length. *P. noxius* was reported from Taiwan as early as 1928 (Sawada 1928). It is conceivable that type M and type S may have existed in Taiwan for a very long period of time and that type M may have evolved in Taiwan earlier and became the predominant type. Only two isolates of type L was obtained from apricot at Taichung City. It is possible that type L may be a recent mutation from subtype M25 through an 6 bp insertion at position 116–117 (V5), and deletion at position 600–601 (V20) (Tables 3, 4). However, the possibility that it may be due to host specificity of type L has not been ruled out.

The results also suggested the possibility that type S may originate from type M through an 8 bp deletion at the position between 135 and 142 (V9) (Tables 3, 4). After the deletion, the ITS sequences seem to become stable because there were only two subtypes among 17 isolates of type S obtained in this study. Moreover, the difference between subtype S1 and subtype S2 was the occurrence of a single nucleotide polymorphism at sequence position 114 (V4) in the latter.

*Phellius noxius* is one of the plant pathogens with a very wide host range. Among the more than 200 plant species representing 59 families listed as hosts of *P. noxius* in the world, about half of them were reported for the first time from Taiwan (Ann et al. 2002). This is compatible with the discovery of great nucleotide variation in ITS region among isolates of *P. noxius* found in Taiwan in this study. The variation included 15 kinds of single nucleotide polymorphisms, three kinds of deletions and two kinds of insertions (Table 3).

Analysis of the ITS sequences of the Taiwanese isolates of *P. noxius* revealed that the 5.8 S region was identical in all isolates, while significant sequence variation was observed in ITS regions. This is in agreement with those reported with powdery mildews (Hirata and Takamatsu 1996) and *Fusarium* species (Naqvi et al. 2013). Our studies showed that the ITS1 was more variable than ITS 2 (Table 3). The former contained 10 single nucleotide polymorphisms, one 8 bp deletion, one 1 bp deletion and one 6 bp insertion, while the latter consisted of only five single nucleotide polymorphisms, one 2 bp deletion and one 1 bp insertion.

Phylogenetic analysis of ITS sequences among *Phellinus* species showed that isolates of *P. noxius* were in the same clade distinctly separated from other *Phellinus* species (Fig. 2). Phylogenetic relationship among *Phellinus* species based on ITS sequences has been reported previously (Shin 2001; Wagner and Fischer 2002; Jeong et al. 2005; Decock et al. 2006). However, none of them has included *P. noxius* in their studies. *P. noxius* has been transferred to *Phellinidium noxium* (Corner) Bondartseva & S. Herrera in 1992 (Bondartseva et al. 1992). However, *Phellinidium noxium* was distinctly separated phylogenetically from other *Phellinidium* species (Dai 2010), indicating that more study is needed in the future.

During this study, *P. noxius* was not found in the counties of Yilan, Taoyuan, Hsinchu and Pingtung (Fig. 1). This does not mean that the fungus was not present in those areas because detection of *P. noxius* in those counties had been reported previously (Ann et al. 2002).

## Conclusion

The 91 isolates of *Phellinus noxius* obtained from 46 plant species in Taiwan showed 20 different kinds of variation including single nucleotide polymorphisms, deletion, insertion in ITS1 and ITS2, but none in 5.8S. The Taiwanese isolates of *P. noxius* were dividable into long (type L), median (type M) and short (type S) groups based on ITS sequence length. Phylogenetic analysis of ITS sequence among *Phellinus* species showed the isolate of *P. noxius* were in the same clade distinctly separated from other *Phellinus* species.
